# *CD40LG*-associated X-linked Hyper-IgM Syndrome (XHIGM) with pulmonary alveolar proteinosis: a case report

**DOI:** 10.1186/s12887-023-04054-6

**Published:** 2023-05-13

**Authors:** Hong-bo Xu, Mao-qiang Tian, Yong-hua Bai, Xiao Ran, Lei Li, Yan Chen

**Affiliations:** 1 Department of Pediatrics, Affiliated Hospital of Zunyi Medical University, No. 143 Dalian Road, Zunyi, 563003 China; 2 Department of Pediatrics, Guizhou Children’s Hospital, Guizhou, 563003 Zunyi China; 3 Department of pathology, Affiliated Hospital of Zunyi Medical University, Guizhou, 563003 Zunyi China

**Keywords:** *CD40LG* gene, X-linked hyper-IgM syndrome, Pulmonary alveolar proteinosis, Pediatrics, Case report

## Abstract

**Background:**

*D40LG*-associated X-linked hyper-IgM syndrome with pulmonary alveolar proteinosis has rarely been reported, and its genotype-phenotypic correlation remains elusive.

**Case presentation:**

We describe a five-month-old boy with *CD40LG* mutation (c.516T > A, p.Tyr172Ter) X-linked hyper-IgM syndrome with pulmonary alveolar proteinosis as the first manifestation. The patient completely recovered after immunotherapy and allogeneic hematopoietic stem cell transplantation. In addition, four previously reported patients with *CD40LG* mutation with pulmonary alveolar proteinosis were also analyzed. All of these patients presented with early onset of pulmonary infections and a good response to immunotherapy. The structural model of *CD40LG* indicated that all mutations caused the X-linked hyper-IgM syndrome with pulmonary alveolar proteinosis to be located within the tumor necrosis factor homology domain.

**Conclusions:**

A case was presented, and the characteristics of four cases of *CD40LG*-associated X-linked hyper-IgM syndrome with pulmonary alveolar proteinosis were summarized. The variant locations may explain the phenotypic heterogeneity of patients with the *CD40LG* mutation.

## Background

The *CD40LG* gene (OMIM* 300,386) encodes the CD40 ligand, which is located on the surface of T cells to regulate B cell function by associating CD40 on the B cell surface. *CD40LG* comprises of four domains: the intracellular domain (IC) in the N-terminal, the transmembrane domain (TM), the extracellular unique domain (EU), and the extracellular tumor necrosis factor homology domain (TNFH) at the C-terminal [1]. A defect of this gene would result in the loss of ability to adjust the immunoglobulin, causing the X-linked hyper-IgM syndrome (XHIGM, OMIM#308,230). XHIGM is a rare group of primary immunodeficiency diseases caused by the disorder of immunoglobulin class conversion, with or without known gene defects. The main characteristic of XHIGM is the significant decrease in blood IgE, IgA and IgG levels, with or without the associated elevated IgM [2]. Pulmonary alveolar proteinosis (PAP) is caused by the lipoprotein-rich alveolar surfactant deposition in the alveolar cavities and airway, resulting in the dysfunction of pulmonary ventilation and gaseous exchange. The typical clinical manifestation of PAP is dyspnea, accompanied by the pulmonary radiograph characteristic of “crazy paving”-like changes [3, 4]. The XHIGM caused by *CD40LG* with PAP as the first manifestation has rarely been reported. The present study summarizes for the first time the clinical features of XHIGM caused by *CD40LG*, and describes an illustrative case which completely recovery achieved after immunotherapy and allogeneic hematopoietic stem cell transplantation (HSCT). Thus, we also analyzed these mutations of *CD40LG*, focusing on any correlations between the phenotypes and molecular subregional locations.

## Case presentation

A five-month-old boy presented with paroxysmal cough and slight wheezing after a cold. The cough did not improve after oral medication and aerosol inhalation treatment at a local clinic. On the contrary, the cough worsened, and was accompanied by dyspnea, cyanosis, wheezing, and elevated work of breathing. The patient was diagnosed with pneumonia accompanied by respiratory failure. Thus, assisted breathing with a ventilator was provided for the patient, and ceftazidime was administered. The wheezes slightly improved after the interventions. However, the respiratory function did not completely improve. Therefore, the patient was transferred to our hospital. The parents of the boy were both non-consanguineous and healthy, and no previous history of genetic disease was declared.

The initial physical examination revealed the following: body temperature of 37.2 °C, pulse of 142/min, breathing at 58/min, 86% percutaneous oxygen saturation (oxygen flow rate, 5 L/min; oxygen inhalation with mask), and weight of 7 kg. The patient’s consciousness was clear with shortness of breath. Furthermore, fine moist rales and wheezes were auscultated in both lungs.

**Fig. 1:**
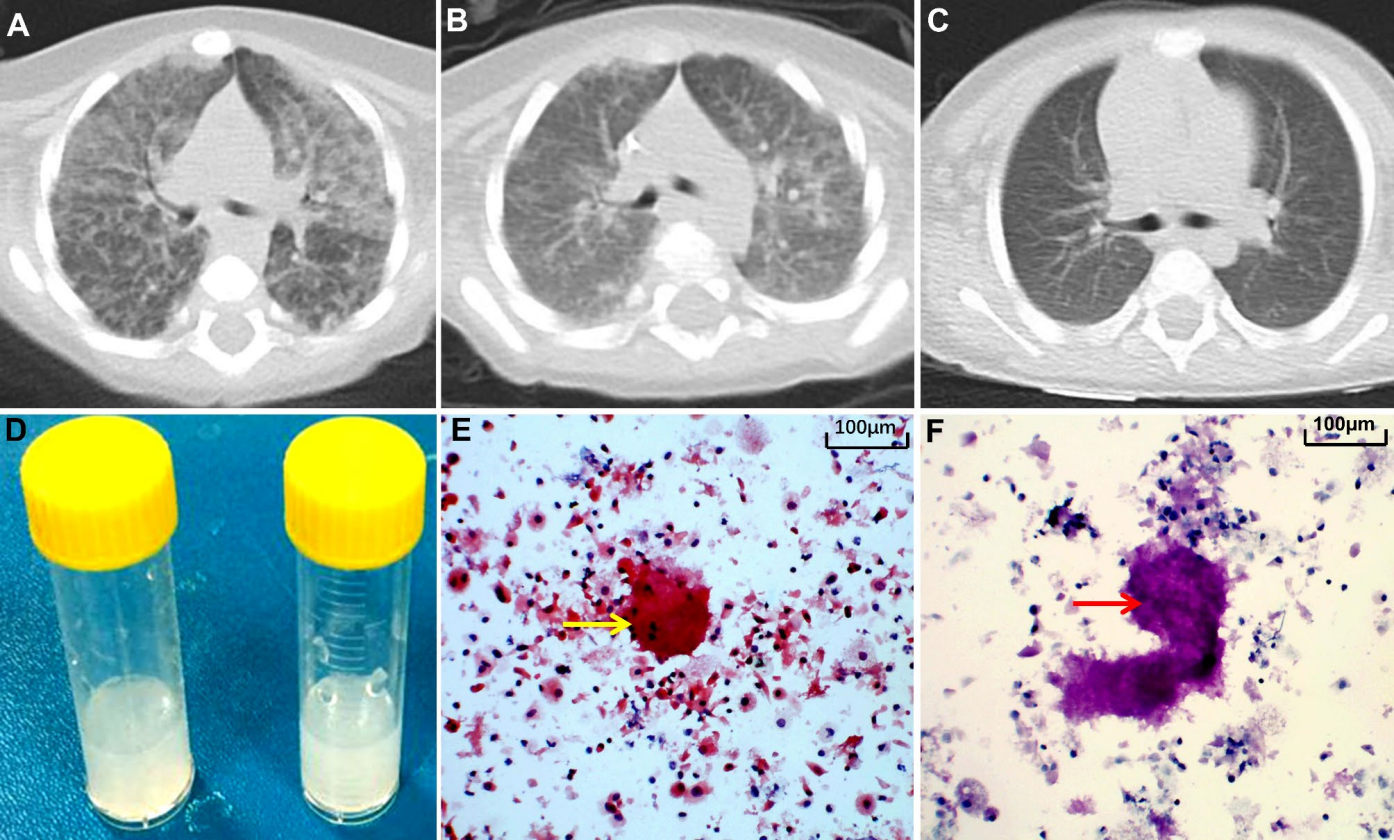
Results for the pulmonary CT and alveolar lavage fluid. (**A**) Pulmonary CT of the patient at admission: “crazy paving” was observed. (**B**) Pulmonary CT at 10 days after admission: the lesion obviously decreased. (**C**) The pulmonary CT revealed that the lesions were almost absorbed after seven months of treatment. (**D**) The milky alveolar lavage fluid. (**E**) The periodate-Schiff reaction (PAS staining) was positive (arrow). (**F**) Positive D-PAS staining (arrow)

The laboratory examination results revealed normal white blood cells (including neutrophils, lymphocytes, monocytes, platelets and red blood cells). However, the high-sensitivity C-reactive protein, procalcitonin, erythrocyte sedimentation rate, and enzyme tests related to the hepatic, renal and myocardial tissues were unremarkable. The serum immunoglobulin tests revealed a significant decrease in IgA and IgG levels, while the IgM level was normal (Table 1). The chest computerized tomography (CT) revealed bilateral diffuse patchy and ground-glass opacities, with a “crazy paving”-like appearance, suggesting bilateral interstitial pneumonia (Fig. 1A). Therefore, the patient was initially diagnosed with interstitial pneumonia with respiratory failure.

**Table 1:** Clinical and genetic characteristics of patients with XHIGM with PAP with the *CD40LG* mutation

Patient no.	Present patient	Patient 2^(6)^	Patient 3^(7)^	Patient 4^(8)^	
**Age of onset (months)**	5	5	12	9	
**Age at diagnosis (months)**	7	5	60	36	
**Delay in diagnosis (months)**	2	0	48	25	
**Predominant infections**	Pneumonia	Pneumonia	Pneumonia/diarrhea	Pneumonia	
**Organisms isolated**	Fungi	No	Klebsiella pneumoniae	Pneumocystis jirovecii	
**Other manifestations**	NA	No	Recurrent anemia	No	
**IgG (mg/dl)**	81 (800–1600)	< 80 (139–655)	308 (490-1 610)	40 (100–560)	
**IgA (mg/dl)**	< 7 (70–330)	8 (4–62)	65 (40–200)	150 (360–920)	
**IgM (mg/dl)**	57 (50–220)	108 (17–69)	314 (50–200)	480 (400–1280)	
**Neutropenia**	Yes	NA	No	Yes	
**Chest CT**	Ground-glass density images in both lungs	Diffuse ground glass opacities, reticular interstitial thickening, and consolidation	Ground-glass density images in both lungs	Ground-glass density images in both lungs	
**Pathology**	BAL material PAS (+)	BAL material PAS (+)	Lung biopsy revealed PAS (+)	BAL material PAS (+)	
**Mutations/domains**	c.516T > A, p.Tyr172Ter/TNFH	c.608G > C, p.Arg203Thr/TNFH	IVS3-1 G > A, E4 skip (del 347–409)/TNFH	c.511 dup A, p. Ile171Asn∗30/TNFH	
**Treatment given and outcomes**	Recovery on WLL, IVIg, cotrimoxazole, and HSCT	Improved on steroids, IVIg and WLL	Improved on IVIg, cotrimoxazole,	Improved on WLL, methylprednisolone, IVIg	
**Follow-up (months)**	42	12	96	36	

**Abbreviations**: BAL, bronchoalveolar lavage; HSCT, hematopoietic stem cell transplantation; IVIg, intravenous immunoglobulin; NA, not available; PAS, periodic acid-Schiff; TNFH, tumor necrosis factor homology domain; WLL, whole-lung lavage

**Fig. 2:**
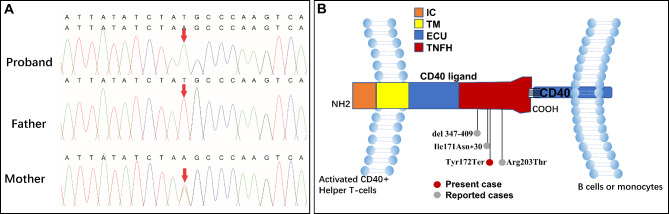
Electropherograms for the Sanger sequencing and schematic diagram of ***CD40LG***. (**A**) The sequencing electropherograms indicate that the mutation *c.516T > A* is from the patient’s mother. (**B**) The intracellular domain (IC), trans-membrane domain (TM), extracellular unique domain (EU), and extracellular tumor necrosis factor homology domain of *CD40LG* are presented in the image. All affected residues/nucleotides in individuals with XHIGM with PAP were located within the TNFH domain

The blood cultures used to identify causative microbes (including mycoplasma, bacteria, tuberculosis and adenovirus) were negative. Fungal infection was suspected due to the G-test result of 195.5 pg/mL (reference value: <10 pg/mL). Pulmonary lesions were remarkably alleviated after the administration of voriconazole (Fig. 1B). On the 11th day after admission, the alveolar lavage fluid was obtained *via* fiberoptic bronchoscopy, which appeared milky (Fig. 1D). The examination results for the periodic acid-Schiff (PAS) stain and diastase-PAS stain were found to be positive in the alveolar lavage fluid (Fig. 1E F), which is consistent with the pathological characteristics of PAP. Hence, the boy was diagnosed with PAP and immunodeficiency. Next, the causes of immunodeficiency and PAP, including the secondary and genetic causes, were further investigated. Polymerase chain reaction and culture tests for *Pneumocystis jirovecii, Mycobacterium tuberculosis*, bacteria, and fungi infection, and the polymerase chain reaction and alveolar lavage fluid culture tests were performed, all results were negative. Trio whole-exon sequencing was subsequently performed using next-generation sequencing (Kangxu Medical Laboratory, Beijing, China). The results identified a *CD40LG* hemizygote nonsense mutation (*NM_000074 c.516T > A, p. Tyr172Ter*). Furthermore, the Sanger sequencing verified that this mutation was inherited from the patient’s asymptomatic mother (Fig. 2A). Thus, this variant was considered to be the cause of the disease, based on the American College of Medical Genetics and Genomics guidelines (PVS1 + PM2) [5]. Finally, the patient was diagnosed with *CD40LG-mutated-* XHIGM with PAP.

**Fig. 3:**
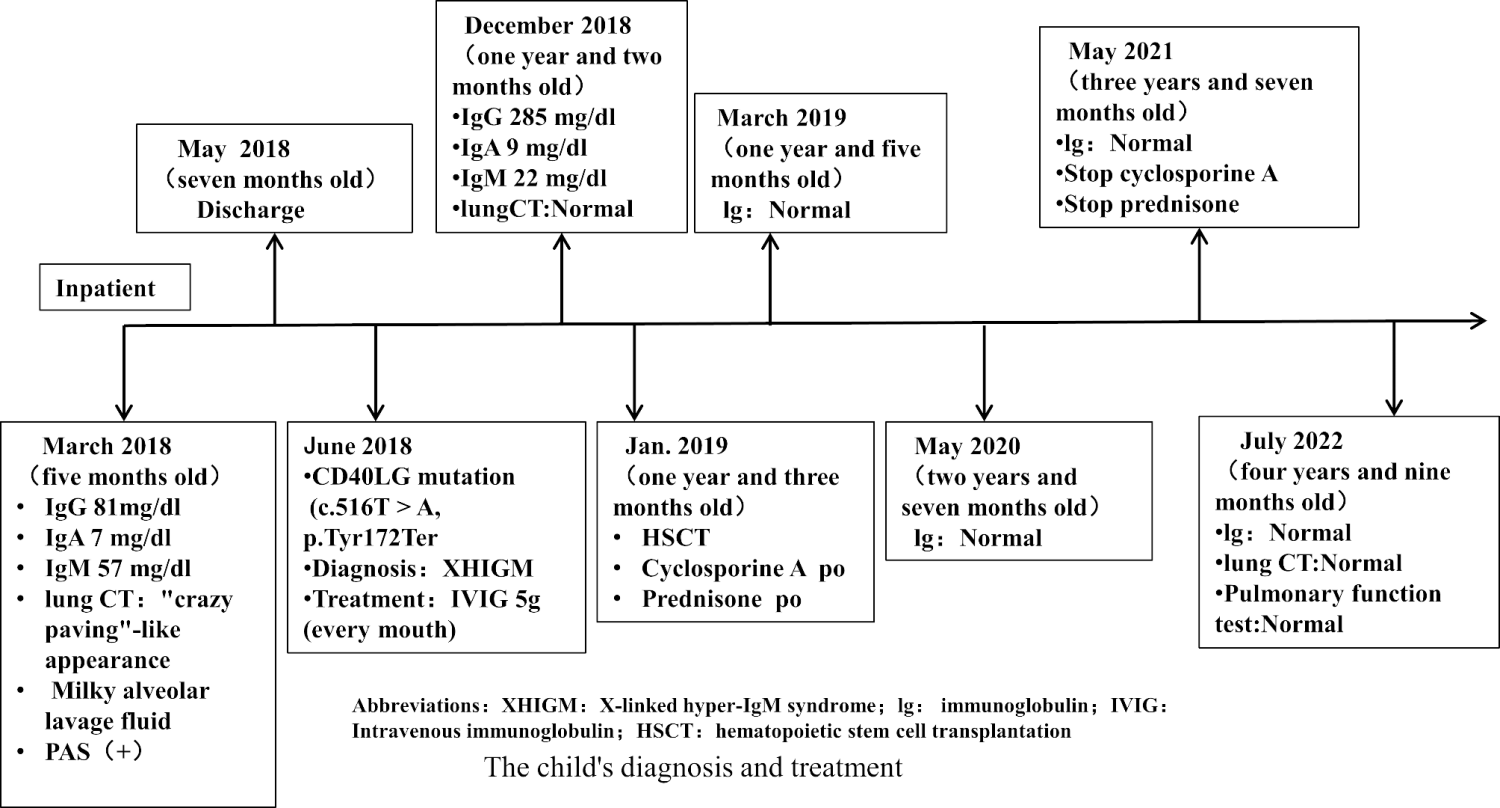
The child’s diagnosis and treatment timeline

A monthly intravenous immunoglobulin infusion combined with oral sulfonamides was administered. The lung lesions completely disappeared after nine months of treatment (Fig. 1C). After obtaining an informed consent, the patient underwent HSCT at the 10th month of the clinical course (HLA match, China Marrow Donor Program, HLA 10/10, donor blood type O/RH^+^, recipient blood type O/RH^+^). The conditioning regimen consisted of busulfan, cyclophosphamide, anti-thymocyte globulin, and graft-versus-host disease prophylaxis with cyclosporine A and methotrexate. The supplementation with immunoglobulin was stopped, because the serum immunoglobulin levels returned to the normal range after myeloid engraftment on day 52. The condition of the patient was good after discharge. The patient was followed up until July 2022 (at the age of four years and nine months old). The patient presented with normal growth and development comparable to the patient’s peers, and had no recurrence of PAP or infection. The serum immunoglobulin and chest CT were also normal. The clinical course of the patient is summarized in Fig. 3.

## Discussion and conclusions

XHIGM represent primary immunodeficiencies presenting with abnormal isotype class switching resulting from damaging mutations in the CD40L/CD40 signaling pathway. As a result, affected patients usually present with a characteristic pattern of normal or elevated IgM and low levels of IgG, IgA, and IgE. Present case and Case 4 showed normal IgM, and Case 2,3 showed elevated IgM, and low levels of IgG, IgA, and IgE [2]. Patients typically present with respiratory tract infections due to common bacterial pathogens and opportunistic infections. XHIGM caused by *CD40LG* with PAP as the first manifestation has rarely been reported, and its genotype-phenotypic correlation remains elusive. The present study presents a case with a novel *CD40LG* (*c.516T > A*, *p.Tyr172Ter*) truncating mutation, XHIGM, with PAP. Combined with the four previously reported patients [6–8], the *CD40LG* mutation XHIGM with PAP was characterized by the early onset of pulmonary infections at approximately the time of infancy (average: 7.75 months old). Most of these patients would experience delays in the diagnosis. The longest misdiagnosis even reached four years. All patients had significantly decreased IgG levels and a good response to immunotherapy. One of these patients (the present case) fully recovered after HSCT. The clinical data of patients with the *CD40LG* mutated XHIGM with PAP are presented in Table 1; Fig. 2B [6–8].

Interstitial pneumonia is not a common complication of XHIGM, only that XHIGM is susceptible to recurrent lung infections or opportunistic infections, and interstitial pneumonia is often one of the imaging features of the aforementioned lung infections. Therefore, when XHIGM patients are combined with interstitial pneumonia, they need to be alert to the possibility of PAP. PAP can be classified into three types: (i) autoimmune PAP, (ii) secondary macrophage-reduced PAP, and (iii) congenital PAP caused by gene mutations, including *SFTPB, SFTPC, ABCA3, GM-CSF2RA*, and *GM-CSF2RB* mutations [3, 9]. The present case report demonstrated that the patient’s PAP was secondary to the immunodeficiency (XHIGM) caused by the *CD40LG* mutation. PAP in XHIGM can be induced by the following pathological causes: (i) Any process that interferes with the production or biological functions of GM-CSF and GM-CSF receptors, or surfactant proteins that can lead to PAP, since one of the important biological functions of macrophages is to catabolize the surfactant within the alveoli [10]. CD40 ligation with CD40L is important for macrophage activation. *CD40LG* gene mutations decrease the scavenging ability of pulmonary macrophages, resulting in the proteinosis of alveolar surfactants (for the present case, the *CD40LG* mutation may have led to GM-CSF dysfunction) [11]; (ii) Humoral immunodeficiency is more likely to create infective opportunities (such as Pneumocystis and fungal infections), which can weaken the phagocytic function of macrophages, and reduce the clearance of the pulmonary surfactant, eventually leading to its overabundance [8, 12]. For the present patient, even though there was no abnormality in the screening of fungi in the alveolar lavage fluid, fungal infection could not be completely excluded, based on the predominant increase in G-test level, and the favorable response to anti-fungal therapy. The PAP of the present patient may have been caused by the combination of *CD40LG* deficiency and infection.

Furthermore, recent studies have revealed that the mutation site at the subregional location of mutations is a critical factor to determine the pathogenicity, and that this is associated with the phenotypic variation [13–15]. *CD40LG* contains the IC, TM, EU and TNFH domains (Fig. 2B). Interestingly, all mutations in the four patients with XHIGM with PAP were located within the TNFH domain. The TNFH domain binds to CD40 on B lymphocytes to trigger the T and B cell interaction, inducing the class switch recombination of the immunoglobulin heavy chain gene [1]. This association can arouse the key signal that produces memory B cells, promoting the transformation of IgM into IgE, IgA and IgG. The function loss of the *CD40LG* gene caused by the gene mutation would result in the non-expression of CD40L on the surface of T lymphocytes. In turn, this would interfere with the T and B lymphocyte association, thereby affecting the transformation of IgM to IgE, IgA and IgG [2, 16]. However, it has been reported that activated T cells expressed normal levels of CD40LG, but presented with decreased CD40-Ig binding, as observed in two patients with the *CD40LG* mutation (*c.608G > T, p.Arg203Ile; c.608G > C, p.Arg203Thr*) that involved the TNFH domain [6, 17]. This evidence suggests that mutations in the TNFH domains may more strongly affect the B cell function. Furthermore, this could explain why the mutations were located in the TNFH domains in all patients with PAP. However, not all mutations in the TNFH domain are associated with PAP. Therefore, more cases need to be accumulated to elucidate the specific causes.

The present study had several limitations. The direct functional effects of the variants were not examined. Furthermore, the total number of the patients with *CD40LG*-associated XHIGM with PAP was relatively small in the present study. Therefore, further studies with more cases are needed to elucidate genotype-phenotype correlation of the *CD40LG* gene.

To our knowledge, the present study is the first to summarize the characteristics of *CD40LG*-associated XHIGM with PAP. The present patient presented with the early onset of pulmonary infections and a good response to immunotherapy. The patient eventually had complete recovery through HSCT. For patients with PAP and immunodeficiency, genetic immunodeficiency should be considered when secondary causes are excluded. Furthermore, the variant locations may explain the phenotypic heterogeneity of patients with the *CD40LG* mutation.

## Data Availability

All supporting data can be made available through the corresponding author upon reasonable request.

## Abbreviations

XHIGM: X-linked hyper-IgM syndrome
PAP: Pulmonary alveolar proteinosis
IC: Intracellular domain
TM: Transmembrane domain
EU: Extracellular unique domain
TNFH: Tumor necrosis factor homology domain
CT: Computed tomography
PAS: Periodic acid-Schiff
HSCT: Allogeneic hematopoietic stem cell transplantation
GM-CSF: Granulocyte colony-stimulating factor
